# Advanced mutant receptor activator of nuclear factor kappa‐Β ligand development with low affinity for osteoprotegerin

**DOI:** 10.1002/ctm2.70195

**Published:** 2025-01-17

**Authors:** Yuria Jang, Yongjin Cho, Youngjong Ko, Yeonhee Moon, Chang‐Moon Lee, Wonbong Lim

**Affiliations:** ^1^ Department of Orthopaedic Surgery Chosun University Gwangju Republic of Korea; ^2^ Laboratory of Orthopaedic Research Chosun University Gwangju Republic of Korea; ^3^ Regional Leading Research Center Chonnam National University Yeosu Republic of Korea; ^4^ Department of Dental Hygiene Chodang University Muangun Republic of Korea; ^5^ School of Healthcare and Biomedical Engineering Chonnam National University Yeosu Republic of Korea; ^6^ Department of Premedical Program School of Medicine Chosun University Gwangju Republic of Korea

1

Dear Editor,

As a new receptor for receptor activator of nuclear factor kappa‐Β ligand (RANKL), Leucine‐rich repeat‐containing G‐protein coupled receptor 4 (LGR4) was recently introduced. Unlike RANKL–RANK signalling, RANKL‐LGR4 inhibits the NFATc1 translocation, thereby inhibiting osteoclastic activity.^1^, ^2^, ^3^ Novel mutant RANKL has been introduced in site‐directed mutations at critical RANKL‐RANK binding sites; it avoids RANK and binds to the LGR4, thereby inhibiting osteoclastogenesis.^4^, ^5^, ^6^ However, a structure similar to that of RANKL has a weakness in the way that it can be scavenged by osteoprotegerin (OPG).^7^ As OPG is an effective substance that removes RANKL, mutant RANKL can also be scavenged.^8^ Therefore, an advanced RANKL mutation was performed for the LGR4 signalling trigger to inhibit osteoclast activity without affinity for OPG, and its applicability as a treatment for osteoporosis was investigated.

The previous mutant RANKL (001) containing transformants at K180R, D189I, R190K, H223F and H224Y and advanced mutant RANKL containing transformants at Q236D (011) or F269L (012), F269Y (013) and F269H (014) in the 011 and were generated for low affinity with OPG in wild type RANKL (WT) (Figure 1A and Figure S1).^9^, ^10^ To determine the candidate with the lowest binding affinity to RANK and OPG (Figures S2 and S3), microscale thermophoresis (MST) was performed between RANK or OPG and mutant RANKL candidates in comparison to wild‐type RANKL (WT) (Figure 1B,C). The binding affinity of 011 and 013 did not show with OPG. The binding affinity between RANK and 011/013 was about 17.1 µM and that between LGR4 and 013 showed the highest (about 8.59 µM and Figure S4). These results indicated 013 to be the potential candidates. To determine which mutant RANKL has the most inhibitory effect on osteoclast differentiation and activities, we stained tartrate‐resistant acid phosphatase (TRAP) and analyzed resorbing pits on mutant RANKL candidates‐ or OPG‐, as a positive control, and treated bone marrow‐derived macrophage cells (BMMs) in the presence of WT. As a result, 013 showed the strongest inhibitory effect on trap‐positive BMMs (Figure 1D,E) and caused a considerable dose‐dependent decrease in TRAP‐positive BMMs (Figure 1F,G). Furthermore, 013 decreased the resorption pit area in WT‐treated BMMs (Figure 1H,I). Therefore, 013 plays an inhibitory role against osteoclast and could be a potential advanced mutant RANKL.

**FIGURE 1:**
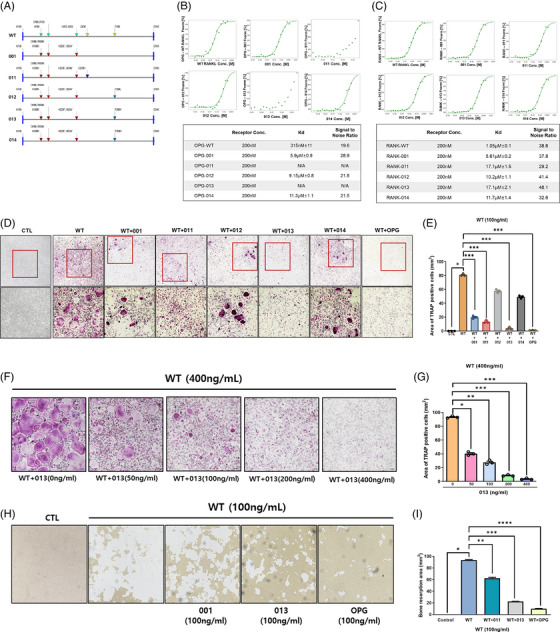
Mutant receptor activator of nuclear factor kappa‐Β ligand (RANKL) and RANK/OPG interaction and Selection of advanced mutant RANKL candidate. (A) Protein sequence of 011 including the K180R, D189I, R190K, H223F, H224Y and Q236D transformants. Protein sequence of 012 including the K180R, D189I, R190K, H223F, H224Y and F269L transformants. Protein sequence of 013 including the K180R, D189I, R190K, H223F, H224Y and F269Y transformants. Protein sequence of 014 including the K180R, D189I, R190K, H223F, H224Y and F269H transformants. (B) The data represent the binding affinities (*K*d values) of osteoprotegerin (OPG) for WT RANKL, 001, 011, 012, 013 and 014. The error bars represent the mean ± S.E. for each data point calculated from three independent thermophoresis measurements. The concentration of WT RANKL, 001, 011, 012, 013 and 014 used in the titration experiments ranged from 11.5 nM to 50 µM, and the concentration of the labelled OPG was constant at 200 nM. (C) The data represent the binding affinities (*K*d values) of RANK for WT RANKL, 001, 011, 012, 013 and 014. (D) Representative images of bone marrow‐derived macrophage cells (BMMs) stained for TRAP (red) after treatment with 001, 011, 012, 013, 014 and OPG in the presence of WT RANKL (100 ng/mL). Magnification represents 20× and 100× in the upper and lower panels, respectively. The scale bar indicates 20 µm. (E) Area of multinucleated TRAP cells (BMM) (red) (≥5 nuclei) in these cultures (*n* = 3). Data are expressed as mean ± SD. *p < .05 for CTL versus WT. ***p < .001 for WT versus WT+001, WT+011, WT+012, WT+013 and WT+014, respectively. (F) A representative image of BMMs stained for TRAP (red) after treatment with various doses of 013 (0, 50, 100, 200 and 400 ng/mL) in the presence or absence of WT RANKL (400 ng/mL). Magnification, 100×. (G) Arear of multinucleated TRAP cells (BMMs) (red) (≥5 nuclei) in these cultures (*n* = 3). Data are expressed as mean ± SD. *p < .05, **p < .01 and ***p < .001 when comparing 013 (0 ng/mL). (F) BMMs were incubated in hydroxyapatite‐coated plates with 001(100 ng/mL), 013 (100 ng/mL), and OPG (100 ng/mL) in the presence or absence of WT RANKL (400 ng/mL). The cells attached to the plate were removed and imaged using a light microscope. (G) The absorption area was quantified using the Image J software. Data are expressed as mean ± SD. *p < .05 for Control versus WT, **p < .05 for WT versus WT + 011 and ***p < .05 for WT versus WT + 013 and ***p < .05 for WT versus WT +OPG.

To investigate the effects of advanced mutant RANKL on the RANK or LGR4 signalling cascade, TRAP staining was performed on BMMs treated with only 013. Compared with WT, 013 did not result in TRAP‐positive BMMs, even after treatment with 400 ng/mL (Figure 2A,B). A marked decrease in TRAP, NFATc1 and OSCAR (markers of osteoclast differentiation and activity) mRNA expressions was noted in 013+WT compared to WT (Figure 2C). The nuclear NFATc1 was not detected in 013‐ or OPG‐treated BMMs in the presence of WT RANKL (Figure 2D,E). Furthermore, AKT and GSK‐3β phosphorylation associated with RANK and LGR4 signalling pathways were performed in WT or 013‐treated BMMs (Figure 2F,G). The AKT phosphorylation between WT and 013 was significantly different in time‐dependent manners. However, no difference between WT and 013 was observed in GSK‐3β phosphorylation. To investigate the effect of advanced mutant RANKL candidate 013 on the RANKL‑induced LGR4 signalling cascade on osteoclastogenesis, we conducted LGR4 conditional knock‐out in RAW 264.7 (RAW264.7/LGR4 CKO) using CRISPR/Cas9 method (Figures S5 and S6). In the presence of WT, the TRAP‐positive area in RAW264.7/LGR4 CKO was increased compared to that in RAW264.7 (Figure 2H,I). In RAW264.7, OPG led to a substantial decrease in AKT phosphorylation and an increase in GSK‑3β in the presence of WT (Figure 2J,K). Furthermore, 013 led to a slight increase of GSK‐3β with or without OPG in RAW264.7 (Figure 2L). No significant difference was noted in GSK‐3β between 013‐ and 013+OPG‐treated RAW264.7/LGR4 CKO. Thus, advanced mutant RANKL could trigger the LGR4‐dependent GSK‐3β signalling in the presence or absence of OPG in the osteoclast precursor cells.

**FIGURE 2:**
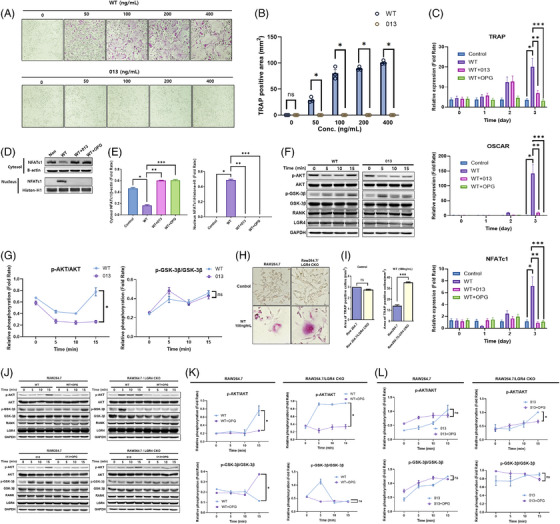
Suppression of osteoclast differentiation and activation by advanced mutant receptor activator of nuclear factor kappa‐Β ligand (RANKL). (A) A representative of bone marrow‐derived macrophage cells (BMMs) stained for TRAP (red) after treatment with various doses of WT RANKL or 013 (0, 50, 100, 200 and 400 ng/mL). Magnification, 100×. (B) Arear of multinucleated TRAP cells (BMMs) (red) (≥5 nuclei) in these cultures (*n* = 3). Data are expressed as mean ± SD. *p < .05 when comparing WT RANKL and 013. N.S., non‐significant (*p* > .05). (C) TRAP, OSCAR, and NFATc1 mRNA expression were analyzed by RT‐PCR. Error bars are mean ± SD. *p < .05 for Control versus WT, **p < .05 for WT versus WT + 013 and ***p < .05 for WT versus WT + osteoprotegerin (OPG). (D) NFATc1 nuclear translocation was analyzed in the cytoplasmic and nuclear fractions. Histone‑H1 and β‑actin were used as loading controls for the nuclear and cytoplasmic fractions, respectively. (E) Densitometric analysis of NFATc1 expression in the cytoplasmic and nuclear fractions presented as the mean ± standard deviation of three separate experiments. *p < .05 for Control versus WT, **p < .05 for WT versus WT + 013 and ***p < .05 for WT versus WT + OPG. (F) Western blots of RANK and LGR4 signalling pathway proteins in WT RANKL (2 µg/mL) or 013 (2 µg/mL)‐treated BMMs. GAPDH was used as a loading control. The results are representative of three separate experiments with comparable results. (G) Densitometric value of p‑AKT/AKT and p‑GSK‑3β/GSK‑3β, as determined by western blot analysis. Results are representative of three separate experiments that had comparable results. *p < .05 for WT versus 013 at 15 min. N.S., non‐significant (*p* > .05). (H) TRAP‐positive multinucleated cells after treatment with WT RANKL in RAW 264.7/*LGR4* CKO and control RAW 264.7 cells. Magnification, 100×. (I) Area of multinucleated TRAP cells (red) (≥5 nuclei) in these cultures (*n* = 3). Data are expressed as mean ± SD. N.S., non‐significant (*p* > .05). *p < .05 when comparing RAW 264.7 and RAW 264.7/*LGR4* CKO cells. (J) Western blots of RANK and LGR4 signalling pathway proteins in WT (2 µg/mL), WT (2 µg/mL) + OPG (2 µg/mL), 013 (2 µg/mL) and 013 (2 µg/mL) + OPG (2 µg/mL)‐treated RAW 264.7 and RAW 264.7/*LGR4* CKO cells. GAPDH was used as a loading control. The results are representative of three separate experiments with comparable results. (K) Densitometric value of p‑AKT/AKT and p‑GSK‑3β/GSK‑3β in WT (2 µg/mL) and WT (2 µg/mL) + OPG (2 µg/mL)‐treated RAW 264.7 and RAW 264.7/*LGR4* CKO cells, as determined by western blot analysis. Results are representative of three separate experiments that had comparable results. *p < .05 for WT versus WT+OPG at 15 min. N.S., non‐significant (*p* > .05). (L) Densitometric value of p‑AKT/AKT and p‑GSK‑3β/GSK‐3β in 013 (2 µg/mL) and 013 (2 µg/mL) + OPG (2 µg/mL)‐treated RAW 264.7 and RAW 264.7/*LGR4* CKO cells, as determined by western blot analysis. *p < .05 for 013 versus 013+OPG at 15 min. N.S., non‐significant (*p* > .05).

To elucidate the effects of advanced mutant RANKL on bone resorption, WT and 013 were administrated to mice (Figure 3A). The trabecular bones from WT+013 were considerably thicker and denser than those from WT (Figure 3B,C). Furthermore, the BV/TV, BV and BMD marks improved. In the case of the cortical bone, Ct.Th. and Ct.Ar did not differ between groups (Figure 3D). Moreover, GST‐RANKL and LGR4 were detected simultaneously in mice inoculated with WT or 013, whereas GST‑RANKL and RANK colocalized only in WT‐induced mice (Figure 3E,F). Therefore, advanced mutant RANKL significantly recovered bone resorption by LGR4 stimulation and suppressed trabecular bone resorption.

**FIGURE 3:**
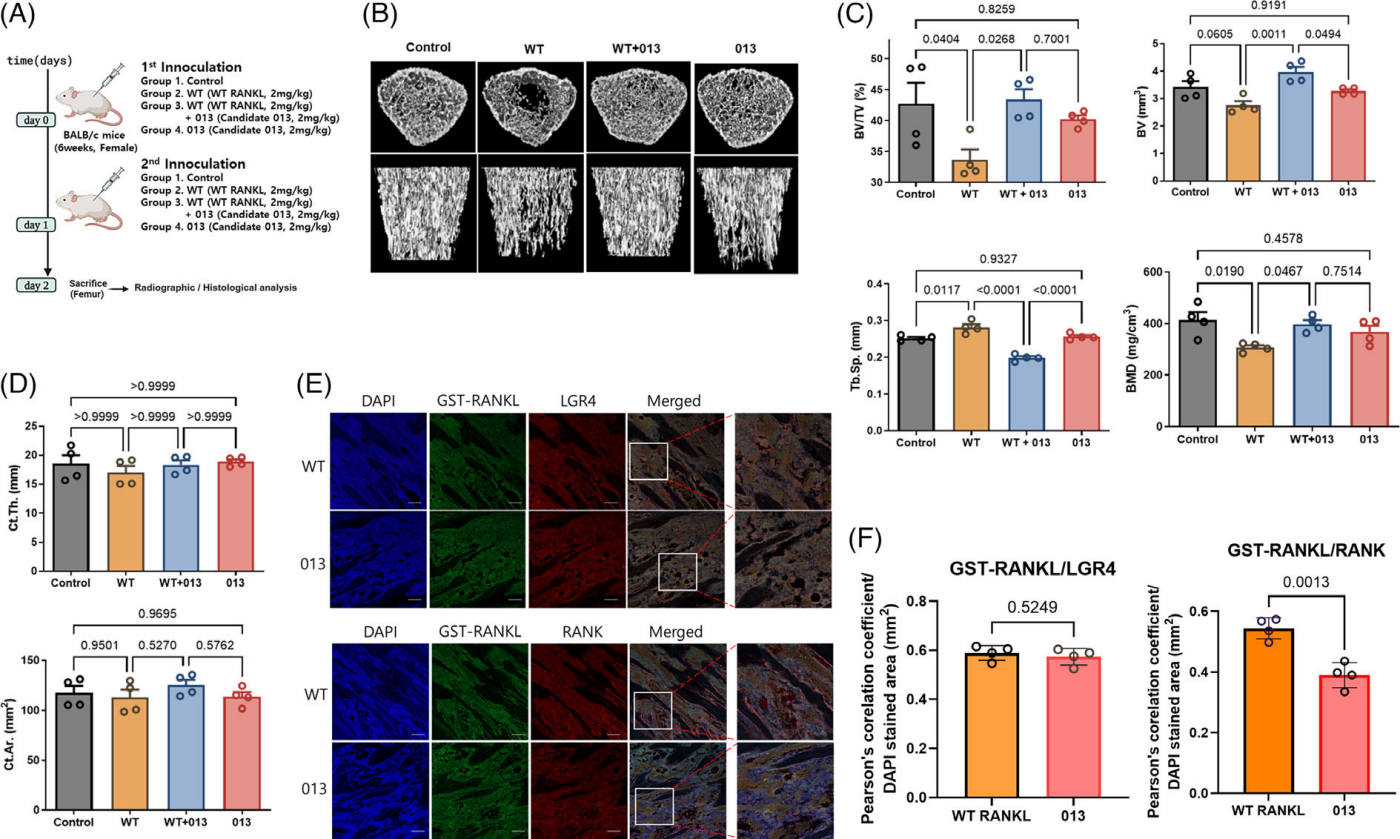
Effect of advanced mutant receptor activator of nuclear factor kappa‐Β ligand (RANKL) on RANKL‐induced mice. (A) Time schedule for inoculation and sampling in Control, WT RANKL (WT)‐, WT RANKL +013‐ (WT +013), and 013‐treated mice. (B) Representative micro‑computed tomography images of the distal femurs of mice. (C) Measurements of bone volume/tissue volume (BV/TV), Bone Volume (BV), trabecular separations (Tb.Sp.), and bone mineral density (BMD). Data are presented as the mean ± SD. *p*‐Values are presented for Control versus WT, WT versus WT RANKL +013, and Control versus 013. (D) Measurements of cortical thickness (Ct.Th.) and cortical bone area (Ct.Ar.). (E) Confocal microscopic images of the co‑localization of GST‑RANKL with LGR4 and RANK in WT RANKL and 013‑treated mice. Magnification, x200; scale bar, 10 µm. (F) Pearson's correlation coefficient was calculated from the merged images of GST/LGR4 and GST/RANK. Data are presented as the mean ± SD of three independent measurements. *p*‐Values are presented for WT RANKL versus 013.

To demonstrate the therapeutic effect of advanced mutant RANKL on bone resorption, OVX mice were inoculated with 013 or OPG (Figure 4A). The trabecular structure of the distal femur revealed that OVX+013 was considerably higher than that of untreated OVX (Figure 4B). In addition, BV/TV and BMD were reduced in OVX mice and recovered by 013, similar to that in OPG mice (Figure 4C,D). Histological analysis using H&E and TRAP staining and immunohistochemical analysis of CTSK showed that OVX mice had enlarged interstitial spaces between the trabecular bone cells (Figure 4E). However, the trabecular bone of OVX+013 showed a thick and dense network with a normal arrangement pattern and minimal space, similar to that in OPG mice. Immunohistochemical analysis of CTSK and TRAP staining results showed that OVX mice had more CTSK‐ and TRAP‐positive osteoclasts than control mice, whereas OVX mice treated with 013 had fewer CTSK‐ and TRAP‐positive osteoclasts than OVX mice (Figure 4F,G). Therefore, advanced mutant RANKL 013 can inhibit OVX‐induced bone resorption.

**FIGURE 4:**
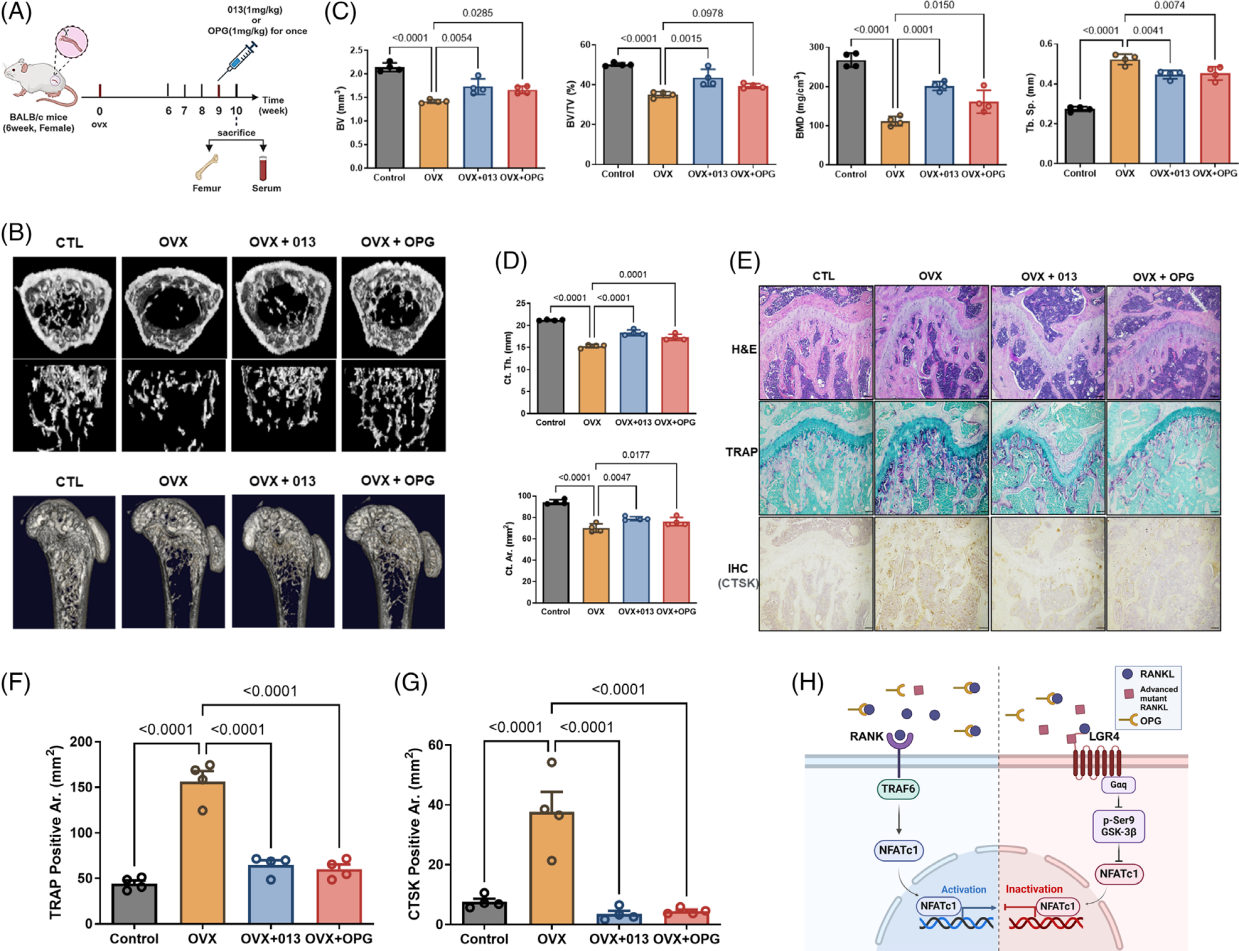
Effect of advanced mutant receptor activator of nuclear factor kappa‐Β ligand (RANKL) on OVX‐induced mice. (A) Time schedule for 013 and osteoprotegerin (OPG) inoculation and sampling in ovariectomized (OVX) mice. (B) Representative micro‐computed tomography images of the distal femurs of mice. (C) Measurements of Bone Volume/Tissue Volume (BV/TV), Bone Volume (BV), Trabecular Separations (Tb.Sp.), and Bone Mineral Density (BMD). Data are presented as the mean ± SD. *p*‐Values are presented for Control versus OVX, OVX versus OVX +013, and OVX versus OVX + OPG. (D) Measurements of cortical thickness (Ct.Th.) and cortical bone area (Ct.Ar.). (E) Hematoxylin and eosin (H&E), Tartrate‐resistant acid phosphatase (TRAP), and Immunohistochemical staining (IHC) of cathepsin K (CTSK) in mice femur. Magnification, ×200; scale bar, 10 µm. (F) Densitometric analysis of TRAP‐positive osteoclast surface area (TRAP‐positive Ar.). (G) Densitometric analysis of Cathepsin K (CTSK)‐positive area (CTSK‐positive Ar.). (H) Schematic diagram of the inhibitory effect of an advanced mutant RANKL against wild‐type RANKL during osteoclastogenesis. To reduce the RANKL‐induced RANK signalling cascade and bone resorption, the effect of advanced mutant RANKL is induced via stimulation of LGR4‐dependent GSK‐3β phosphorylation without OPG binding.

The present study has several limitations. First, limited validations exist on the effects of RANK signalling cascades or bone formation for balancing bone homeostasis. Second, the effect of long‐period treatments on mice models may be required for further translational study Supporting Information.

Finally, the advanced mutant RANKL 013 which contains five‐point mutations at the RANK binding site (K180R, D189I, R190K, H223F and H224Y) and one‐point mutation at affecting OPG binding (F269Y), appears to inhibit osteoclast activation through LGR4 signalling stimulations without obstruction by exogenous OPG (Figure 4H). Therefore, developing an advanced mutant RANKL could be an innovative therapeutic strategy against osteoporosis.

## AUTHOR CONTRIBUTIONS

Yuria Jang, Yongjin Cho, Youngjong Ko, Yeonhee Moon, Chang‐Moon Lee, and Wonbong Lim designed and performed the research, analyzed, and interpreted data and wrote the paper; Yuria Jang and Yongjin Cho performed in vitro and in vivo studies under the supervision of Wonbong Lim; Chang‐Moon Lee performed Micro‐CT analysis and contributed to some of the figures; Yeonhee Moon performed a pathological review of samples; Wonbong Lim analyzed publicly available datasets and supervised the study.

## CONFLICT OF INTEREST STATEMENT

The authors declare no conflict of interest.

## FUNDING INFORMATION

This study was supported by the National Research Foundation of Korea (NRF) and was funded by the Korean government (NRF‐2021R1A6A3A13046475, NRF‐2022R1A2C2009119, NRF‐2021R1I1A3046499 and RS‐2023‐00217471).

## ETHICS STATEMENT

All animal experiments were carried out in compliance with institutional and governmental requirements approved by the Institutional Animal Care and Use Committee (approval no. CIACUC2022‑S0008) of Chosun University, Gwangju, South Korea.

## Supporting information



Supporting Information

Supporting Information

## Data Availability

All requests for raw and analyzed data and materials will be promptly reviewed to verify whether the request is subject to any intellectual property or confidentiality obligations by the corresponding author and Chosun University, Republic of Korea.
