# Supplementary material for: Advanced mutant receptor activator of nuclear factor kappa‐Β ligand development with low affinity for osteoprotegerin
Source: Clin Transl Med. 2025 Jan 17;15(1):e70195. doi: 10.1002/ctm2.70195 (PMC11740215; doi:10.1002/ctm2.70195)
Supplement: Supplementary file 1 — Supporting Information [file CTM2-15-e70195-s001.docx]

Supplementary materials and methods

**Advanced mutant RANKL development with low affinity for OPG**

Yuria Jang, Yongjin Cho, Youngjong Ko, Yeonhee Moon, Chang-Moon Lee, **Wonbong Lim**

**This file includes:**

Supplementary materials and methods

**Advanced mutant RANKL generation**

Wild type (WT) and advanced mutant RANKL were generated as previously described ^1^. Briefly, plasmids carrying WT and mutant RANKL were expressed in *Escherichia coli* BL21-Gold. The genes with mutations at RANK-binding amino acid positions 180, 189, 190, 223, 224, 236, and 269 in mouse RANKL, which were matched with human RANKL at positions 181, 190, 191,224, 225, 237, and 270, were generated by Bioneer Co. (Daejeon Korea), and the product was cloned into the NdeI/XhoI site of the pGEX-4T-1 vector (Promega, Madison, WI). The protein product was purified as previously described ^1^. The previous mutant RANKL (001) containing transformants at K180R, D189I, R190K, H223F, and H224Y and the advanced mutant RANKL containing transformants at Q236D (011) or F269L (012), F269Y (013), and F269H (014) in the 011 were generated with low affinity for OPG in wild type RANKL (WT).

**RANK, LGR4 and OPG generation and purification**

The genes encoding RANK, OPG and LGR4 were synthesized using GeneArt gene synthesis (Thermo Fisher Scientific, Waltham, MA, USA) and then subcloned into the pETM-13 vector (EMBL, Heidelberg, Germany) as previously described ^1^. The recombinant RANK, OPG and LGR4 protein were expressed in E. coli BL21(DE3) cells. The genes encoding RANK and LGR4 were synthesized and codon-optimized for Escherichia coli expression by GeneArt Gene Synthesis (Thermo Fisher Scientific, Inc.), and then subcloned into the pETM-13 vector (EMBL) between the NcoI and XhoI restriction sites. This plasmid enables a histidine (His) tag to be placed at the C-terminus of the mini-protein. The recombinant protein was successfully overexpressed in E. coli BL21(DE3) cells (Thermo Fisher Scientific, Inc.), resulting in a yield of 70 mg/l. Briefly, an overnight starting culture of 10 ml was prepared for growth in 1 l of Luria Bertani medium containing kanamycin (50 µg/l), which was then induced with isopropyl β-D-1-thiogalactopyranoside (0.8 mM) at 16˚C for 16 h. The protein was purified by ultrasonicating the bacterial cells at 20 KHz, at 5˚C for 15 min, which were then resuspended in binding buffer [300 mM NaCl, 50 mM Tris-HCl, 10 mM imidazole, 2.5% (v/v) glycerol, pH 7.8] containing a protease inhibitor cocktail (Roche Diagnostics). The cell lysate was then centrifuged for 30 min at 14,000 x g and 5˚C to remove the cell debris and inclusion bodies. The supernatant was centrifuged again for 30 min at 40,000 x g and 5˚C after which, the resulting membrane pellet was resuspended in PBS lysis buffer [PBS, pH 8.0; 10% (w/v) glycerol, 1 mM DTT, 0.002% (w/v) phenylmethylsulfonyl fluoride (PMSF) and 10 mg/l DNase I (PanReac AppliChem); 5 ml PBS lysis buffer per 1 g cells] and the cell membranes were isolated by centrifugation at 34,000 x g and 5˚C for 30 min. The membrane pellets were flash-frozen in liquid nitrogen and stored at -80˚C. To solubilize the protein, the membranes were resuspended in buffer S [50 mM Tris/HCl, pH 7.8; 200 mM NaCl; 1.2% (w/v) FosCholine-16; 2.5 mM DTT; 0.002% (w/v) PMSF] and stirred at 700 rpm for 1 h. The cell lysate was ultracentrifuged at 230,000 x g and 5˚C for 60 min. The resulting supernatant was loaded onto a 5 ml Ni-NTA HisTrap HP column (Cytiva) equilibrated in buffer C-P [buffer C (50 mM HEPES/NaOH, pH 7.6; 300 mM NaCl, 5% (w/v) glycerol; 5 ml lysis buffer C per 1 g cells) with 0.002 % [w/v] PMSF). The column was washed with 10 column volumes (CV) of buffer C-P, followed by 20 CV of buffer C-ATP [buffer C with an additional 50 mM KCl, 20 mM MgCl2, 10 mM ATP, and 0.002% (w/v) PMSF]. The column was further washed with 10 CV of buffer C-P containing 50 mM imidazole and 100 mM imidazole. The RANK and LGR4 proteins were eluted with 500 mM imidazole in the C-P buffer. The purified proteins were concentrated in a 50 kDa Amicon Ultra-15 concentrator (MilliporeSigma). The buffer was changed by desalting on a PD-10 column (Cytiva) for binding affinity measurement.

**Binding affinity measurement**

The protein-binding affinity was measured using microscale thermophoresis (MST), as previously described ^2^. The MST experiments were performed using Monolith NT.115 systems (NanoTemper Technologies GmbH) and a red filter. Briefly, all dilutions were prepared to ensure that no other gradient (salt, glycerol, DMSO, etc.) was created during the buffer mixing. To minimize the adsorption of the sample to the material, 0.05% Tween 20 was added to PBS, which was used to dilute all of the receptors and ligands. To measure protein-protein binding, the receptor protein RANK, OPG or LGR4, and RANKL ligands were mixed with an equal volume of the fluorescent ligand spiperone-Cy5 (NanoTemper Technologies GmbH) to obtain final ligand concentrations of 0.125, 5, 7.5 and 12 nM. After incubation at 20 ˚C for 1 h, the samples were loaded onto capillaries and the LED was set to 20% for 0.125 nM samples, and 1% for 5, 7.5 and 12 nM samples, using medium MST power. For the receptor titration assay, various concentrations of protein (10 mg/ml-5 µg/ml total protein) were mixed with a specific concentration of the fluorescent ligand, namely, spiperone-Cy5 was added at a final concentration of 5, 7.5 or 12 nM to each protein dilution point. The sample was incubated at 20 ˚C for 1 h before capillary loading. The LED power was set to 1% and the MST power was set to medium. The intersection points in the binding curves were determined according to the manufacturer’s protocol and equilibrium dissociation constant (Kd) values were obtained using Frobenius normalization (%) from the mean of three replicates.

**TRAP assay**

All of the in vitro and in vivo experimental procedures involving mice study were performed in compliance with institutional and governmental requirements, and were approved by the Institutional Animal Care and Use Committee (approval no. CIACUC2022‑S0008) of Chosun University (Gwangju, Republic of Korea). For TRAP assay, bone marrow cells (BMCs) were obtained according to previous protocol ^3^. For RAW 264.7 cells, seeding was performed using RAW 264.7 and RAW264.7/LGR4 CKO cells on the first day, medium replacement was performed on the third day, and fixation and staining were performed on the fifth day. After three washes with distilled water, TRAP staining was performed for 30-40 min according to the manufacturer’s instructions (Kamiya Biomedical Co.). The stained cells were examined under an ECLIPSE Ts2R inverted light microscope (Nikon Corporation) and images were captured using a digital camera (Nikon Corporation) with NIS-Elements imaging software (Nikon Corporation). The TRAP-positive area was identified as OC and measured using Image J 1.52a software (National Institutes of Health).

**Bone resorption assay**

To observe bone resorption in vitro, BMCs were cultured to Corning® Osteo Assay Surface 96-well Multiple Well Plates (Sigma, Cat no. CLS3988) for 6 days. The plates were then washed with pure water. The images of bone resorbing area were acquired using an ECLIPSE Ts2R inverted microscope (Nikon) and measured using Image J 1.52a software.

**Western blot analysis**

To assess the protein expressions, the western blot analysis was performed according to previous study^3^. Briefly, 30 mg of the cell lysates were separated by 10% SDS-PAGE and transferred onto a polyvinylidene difluoride (PVDF) membrane (Amersham, Piscataway, NJ, USA). The membrane was then incubated with the appropriate primary antibodies, namely p-Akt (1:1000; 9271S; Cell Signaling Technology), Akt (1:1000; 9272S; Cell Signaling Technology), p-GSK-3β (1:1000; 9336S; Cell Signaling Technology), GSK-3β (1:1000; 9315S; Cell Signaling Technology), RANK (1:1000; 4845S; Cell Signaling Technology), LGR4 (1:1000; PA5-109908; Invitrogen), NFATc1 (1:1,000; cat. no. 8032; Cell Signaling Technology, Inc.), Histone H1 (1:1,000; cat. no. sc‑393358; Santa Cruz Biotechnology, Inc.), and β‑Actin (1:1,000; cat. no. sc‑47778; Santa Cruz Biotechnology). A mouse monoclonal immunoglobulin G antibody specific for GAPDH (1:1,000; cat. no. 97166; Cell Signaling Technology, Inc.) was used as the control. The blot images were acquired using a chemiluminescence imaging system (Vilber Lourmat) and the densitometric analysis of the bands was carried out using Image J 1.52a software after normalization to GAPDH.

**Separation of nuclear and cytoplasmic fractions for NFATc1 detection**

The BMCs were incubated with 75 ng/ml WT or/and 75 ng/ml 013 or 75 ng/ml OPG for 24 h in 6-well plates. The cells were rinsed in PBS and collected in microtubes (Eppendorf). A 0.5-ml volume of Solution A (10 mM HEPES, 1.5 mM MgCl_2_, 10 mM KCl, 0.5 mM DTT, 0.05% NP40, pH 7.9) was then added, after which the cells were centrifuged at 805 x g for 10 min at 4˚C. The supernatant, containing mostly cytoplasmic constituents, was then removed and transferred to another tube. To yield a nuclear pellet, 0.4 ml solution B [5 mM HEPES, 1.5 mM MgCl2, 0.2 mM EDTA, 0.5 mM DTT, 26% glycerol (v/v), 300 mM NaCl, pH 7.9] was added and the contents of the tube were mixed thoroughly and placed on a small rotator shaker for 15 min. Finally, the mixture was centrifuged at 24,000 x g for 20 min at 4˚C. The supernatant containing proteins from the nuclear extract was removed and transferred carefully into a fresh tube. The nuclear and cytosolic extracts were frozen at -80˚C in aliquots prior to western blot analysis. The protein concentration of each sample was determined using the BCA protein assay kit (Thermo Fisher Scientific, Inc.), and the cytosolic and nuclear fractions were subjected to western blot analysis. Histone-H1 and β-actin were used as loading controls for the nuclear and cytoplasmic fractions, respectively.

**Real-time reverse transcription PCR (RT-PCR) analysis**

The BMCs were incubated with 30 ng/ml M-CSF and 75 ng/ml WT, or/and 75 ng/ml 013 or 75 ng/ml OPG at 37.5˚C for the indicated times (0, 1, 2 and 3 days) in 6-well plates. Total RNA was extracted from the cells using TRIzol® reagent (Invitrogen; Thermo Fisher Scientific, Inc.). The cDNA was then obtained from 2 μg total RNA using ReverTra Ace qPCR RT master mix (Toyobo Life Science) according to the manufacturer’s protocol. The mRNA expression levels were measured using qPCR and GAPDH was used as a control. qPCR was performed with a CFX Connect real-time PCR detection system (Bio-Rad Laboratories, Inc.) using a 20 μl reaction mixture containing 10 μl IQ SYBR Green supermix (Bio-Rad Laboratories, Inc.), 10 pmol forward primer, 10 pmol reverse primer and 1 μg cDNA. The sequences of the primers used to target TRAP, OSCAR, NFATc1 and GAPDH are presented as follows: TRAP, 5’-TACCTGTGTGGACATGACC-3’ (forward) and 5’-CAGATCCATAGTGAAACCGC-3′ (reverse); OSCAR, 5′-CTGCTGGTAACGGATCAGCTCCCCAGA-3′ (forward) and 5′-CCAAGGAGCCAGAACCTTCGAAACT-3′ (reverse); NFATc1, 5′-GAGTACACCTTCCAGCACCTT-3′ (forward) and 5′-TATGATGTCGGGGAAAGAGA-3′ (reverse) and GAPDH, 5′-TCAAGAAGGTGGTGAAGCAG-3′ (forward) and 5′-AGTGGGAGTTGCTGTTGAAGT-3′ (reverse).The amplification parameters consisted of an initial denaturation step at 95.8˚C for 5 min followed by 40 cycles of three-step PCR (denaturation at 95.8˚C for 1 min, annealing at 60.8˚C for 30 sec and extension at 72.8˚C for 1 min). The fluorescence resulting from the incorporation of SYBR Green dye into the double-stranded DNA produced during PCR was quantified using the 2-ΔΔCq method ^4^.

**Generation of RAW264.7/LGR4^-/-^ cells**

The CRISPR/Cas9 method was used to knockout LGR4 from the RAW 264.7 cell line and generate RAW264.7/LGR4^-/-^ cell lines. The cell line was produced by Ubigene (Guangzhou, China). Because the LGR4 gene is large, the entire deletion was not performed, and the middle of exon 5 was cut to induce a mutation that would inactivate the sequence. For Lgr4 KO, single guide RNAs (sgRNAs) were designed using the online CRISPR design tool (Red CottonTM, Guangzhou, China, https://en.rc-crispr.com/). The exon 5 region of Lgr4 was selected for CRISPR/Cas9 genome editing. A ranked list of sgRNAs was generated using the specificity and efficiency scores. The pair of oligos for the two target sites was annealed and ligated into a YKO-RP003 vector (Ubigene Biosciences Co., Ltd., Guangzhou, China). YKO-RP003-mLgr4[gRNA3/4] plasmids containing each target sgRNA sequence were transfected into cells using the Neon Transfection System (Invitrogen). Twenty-four hours after the transfection, puromycin was added to screen the cells. After antibiotic selection, cells were diluted using the limited dilution method and inoculated into a 96-well plate. Single clones were performed after 2–4 weeks and the selected Lgr4 KO clones were validated by PCR and Sanger sequencing. The sgRNAs for CRISPR design are as follows: LGR4 CKO-F; 5’-GCTTAGATGCCAACCATATTACCTCAG-3’, LGR4 CKO-R; 5’- TTGTTATGAAGATGCAGCACTACCAA-3’.

**PCR of genomic DNA**

PCR was performed using RAW 264.7 and LGR4 CKO RAW 264.7 cells. gDNA was extracted using the Accuprep Genomic DNA Extraction Kit (BIONEER, K-3032). PCR was performed using the TaKaRa LA Taq® DNA polymerase (TaKaRa, RR002A). A total of 40 cycles were used with initial denaturation at 95 °C for 5 min, denaturation at 95 °C for 20 s, annealing at 58.1°C for 15 s, and extension at 72 °C for 15 s. Mouse GAPDH was used as a loading control, and mouse LGR4 exon5 was used to detect LGR4. The sequences of the primers used are as follows: LGR4 CKO-F; 5’- GCTTAGATGCCAACCATATTACCTCAG -3’, LGR4 CKO-R; 5’- TTGTTATGAAGATGCAGCACTACCAA -3’.

**Mouse inoculation and ovariectomy**

Six-week-old female mice (BALB/c; Orient Bio Co. LTD, Seoul, South Korea) were housed under controlled 12 h light/dark cycle conditions and fed ad libitum. For RANKL injection, 20 mice were equally divided into four groups. The control group was injected intraperitoneally with PBS, the WT RANKL group received WT RANKL (2 mg/kg) in PBS, the WT RANKL + 013 group received 013 (2 mg/kg) along with WT RANKL (2 mg/kg) in PBS, and the 013 group received 013 (2 mg/kg) was administered only 013 (2 mg/kg) in PBS at 24-h intervals for 2 days. The mice were sacrificed on day 3 according to the indicated schedule.

To investigate ovariectomized mice, twenty mice were equally divided into four groups. Group 1 (Control group) received an intraperitoneal injection of PBS, and group 2 (OVX group) was ovariectomized and injected with PBS. Group 3 (OVX+013) was ovariectomized and injected with 013 (1 mg/kg). In group 4 (OVX+OPG), the ovaries were removed and injected with OPG. Mice were sacrificed after 10 weeks. Finally, femur bone and blood samples were collected for further analysis.

**Micro-computed tomography (CT) imaging and data acquisition**

Micro-CT scanning of the distal femur was initiated at the level of the growth plate using a Quantum GX micro-CT imaging system (PerkinElmer, Hopkinton, MA, USA) located at the Korea Basic Science Institute (KBSI) in Gwangju, Korea, according to previous study ^1^. The bone volume of the tissue volume (BV/TV), trabecular separation (Tb. Sp.) and bone mineral density (BMD) of the femurs were calculated using the ROI tool. Parameters values are shown as mean ± standard deviation (SD).

**Histological analysis of mouse tissues**

Mouse femurs were dissected, immersed in 4% formaldehyde, and decalcified in 7% EDTA with 0.5% paraformaldehyde for 30 days before processing. To analyze longitudinal sections of distal femurs, decalcified tissues were paraffin-embedded, and 3-μm-thick sections were cut, mounted on glass slides, and rehydrated using graded alcohol. Tissue sections were stained with hematoxylin/eosin (Shandon Varistain 24-4, Histocom, Vienna, Austria), and images were acquired using an ECLIPSE Ts2R inverted microscope (Nikon).

**Immunofluorescence analysis of bone specimens**

Paraffin-embedded bone sections were prepared according to the aforementioned protocol and incubated overnight at 4˚C with the following primary antibodies: Anti‑LGR4 (1:100; cat. no. PA5‑67868; Invitrogen; Thermo Fisher Scientific, Inc.), ani‑RANK (1:100; cat. no. PA5‑88904; Invitrogen; Thermo Fisher Scientific, Inc.), and anti-glutathione S-transferase (GST; 1:100; cat. no. 13‑6700; Invitrogen; Thermo Fisher Scientific, Inc.). Subsequently, sections were stained at 4˚C for 1 h using Alexa Fluor 594 goat anti‑rabbit (1:500; cat. no. A11037; Invitrogen, Thermo Fisher Scientific, Inc.) and Alexa Fluor 594 goat anti–mouse secondary antibodies (1:500; cat. no. A11032; Invitrogen; Thermo Fisher Scientific, Inc.). After washing with PBST, the immunolabeled cells were counterstained with DAPI in Pro-Long Gold mounting solution (Invitrogen; Thermo Fisher Scientific, Inc.). Digital images were acquired using a TCS SP5 AOBS laser‑scanning confocal microscope (Leica Microsystems GmbH) and co‑localization of RANKL and LGR4, or RANKL and RANK, was analyzed by comparing the Pearson correlation coefficient between RANKL‑treated groups.

**TRAP staining of mouse tissues**

To evaluate TRAP activity, tissue sections were deparaffinized, rinsed with TBS, and incubated with a solution containing 50 mM sodium acetate (pH 5.2), 0.15% Naphthol-AS-TR-phosphate, 50 mM sodium tartrate, and 0.1% Fast Red T.R. (Sigma Aldrich Chemie Gmbh, Taufkirchen, Germany) for 30–40 min at room temperature. The sections were then rinsed with TBS and counterstained with 0.2% methyl green. Finally, the images were acquired using an ECLIPSE Ts2R inverted microscope (Nikon).

**Immunohistochemical analysis of bone specimens**

For the immunohistochemical study of bone specimens, the slides were prepared according to previous protocol ^1^. Negative and positive controls were analyzed simultaneously. Slides were imaged using an inverted microscope (Nikon) and immune-positive area was analyzed using Image J software.

**Statistical analysis**

All *in vitro* and *in vivo* experiments were conducted at least in triplicate. All quantitative results are presented as the mean ± SD. The primary comparisons of cell‑based data and data from all the animal studies were analyzed using a one‑way or two‑way analysis of variance with a Bonferroni multiple‑comparisons test or unpaired Student's t‑test. All reported P‑values were two-sided, and P<0.05 was considered to indicate a statistically significant difference. All statistical analyses were performed using GraphPad Prism version 7 (Dotmatics).

**REFERENCES**

1. Ko YJ, Sohn HM, Jang Y*, et al.* A novel modified RANKL variant can prevent osteoporosis by acting as a vaccine and an inhibitor. *Clin Transl Med.* 2021; 11: e368.

2. Jang Y, Lee H, Cho Y*, et al.* An LGR4 agonist activates the GSK‑3beta pathway to inhibit RANK‑RANKL signaling during osteoclastogenesis in bone marrow‑derived macrophages. *Int J Mol Med.* 2024; 53.

3. Jang Y, Sohn HM, Ko YJ, Hyun H, Lim W. Inhibition of RANKL-Induced Osteoclastogenesis by Novel Mutant RANKL. *Int J Mol Sci.* 2021; 22.

4. Livak KJ and Schmittgen TD. Analysis of relative gene expression data using real-time quantitative PCR and the 2(-Delta Delta C(T)) Method. *Methods, 2001*; 25: 402-408.
