# Supplementary material for: Advanced mutant receptor activator of nuclear factor kappa‐Β ligand development with low affinity for osteoprotegerin
Source: Clin Transl Med. 2025 Jan 17;15(1):e70195. doi: 10.1002/ctm2.70195 (PMC11740215; doi:10.1002/ctm2.70195)
Supplement: Supplementary file 2 — Supporting Information [file CTM2-15-e70195-s002.docx]

Supplementary figures

**Advanced mutant RANKL development with low affinity for OPG**

Yuria Jang, Yongjin Cho, Youngjong Ko, Yeonhee Moon, Chang-Moon Lee, **Wonbong Lim**

**This file includes:**

Figure S1 - S12


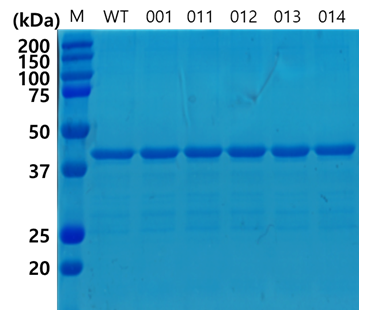
S.1 The previous mutant RANKL (001) containing transformants at K180R, D189I, R190K, H223F, and H224Y and the advanced mutant RANKL containing transformants at Q236D (011) or F269L (012), F269Y (013), and F269H (014) in the 011 were generated with low affinity for OPG in wild type RANKL (WT)


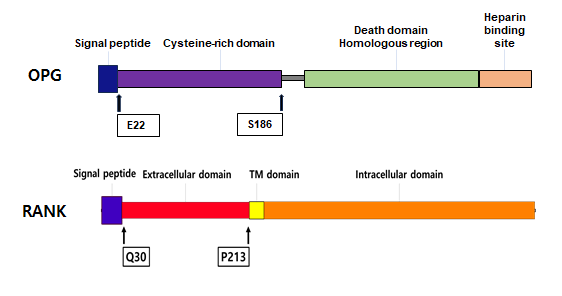


S.2 The OPG and RANK receptor proteins used in the present study. The extracellular domains of OPG and RANK as a receptor protein for ligand- binding affinity measurements are shown. The OPG sequence from E22 to S186 is the cysteine-rich domain that represents the ligand-binding domain. The ligand-binding domain of RANK is from Q30 to P213.


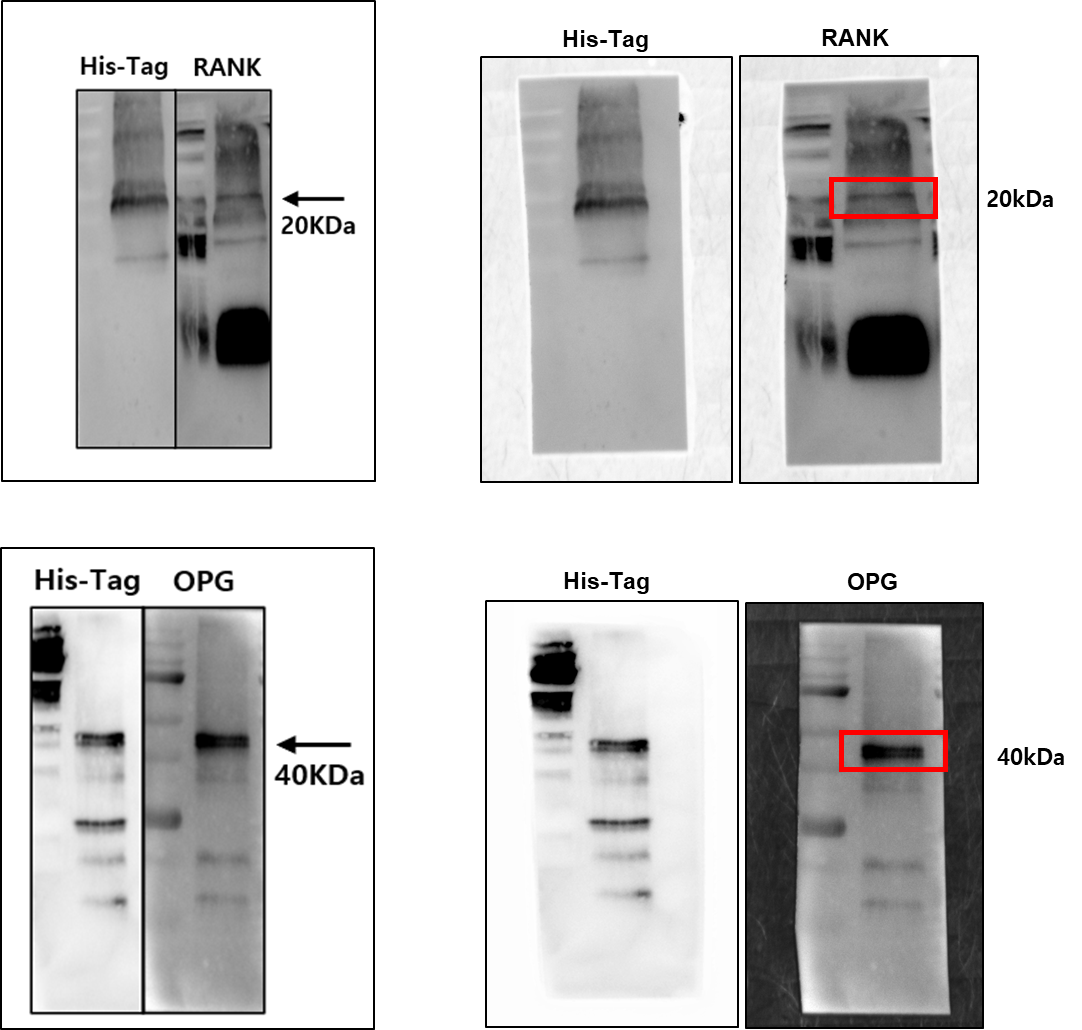


S.3 Original western blot analysis of His-tagged fusion proteins (OPG, RANK). Purified receptor proteins were identified by western blot analysis. His-tagged OPG and RANK were shown at 40 and 20 KDa, respectively.


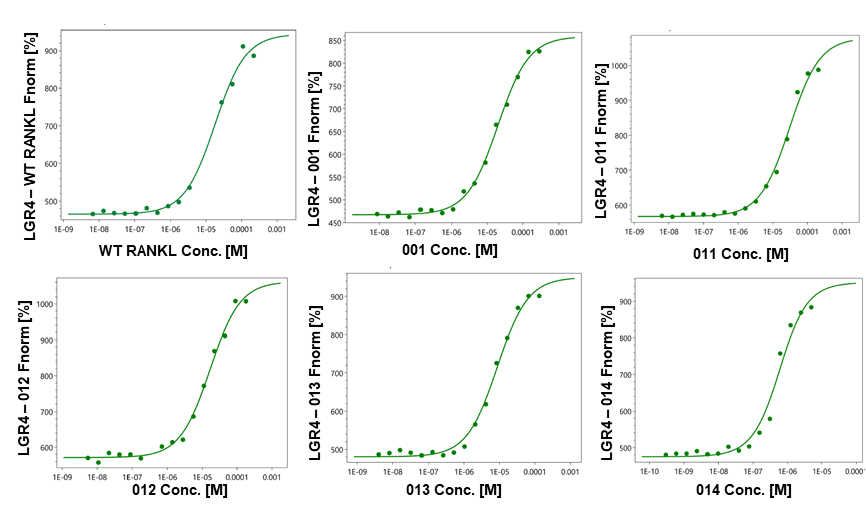


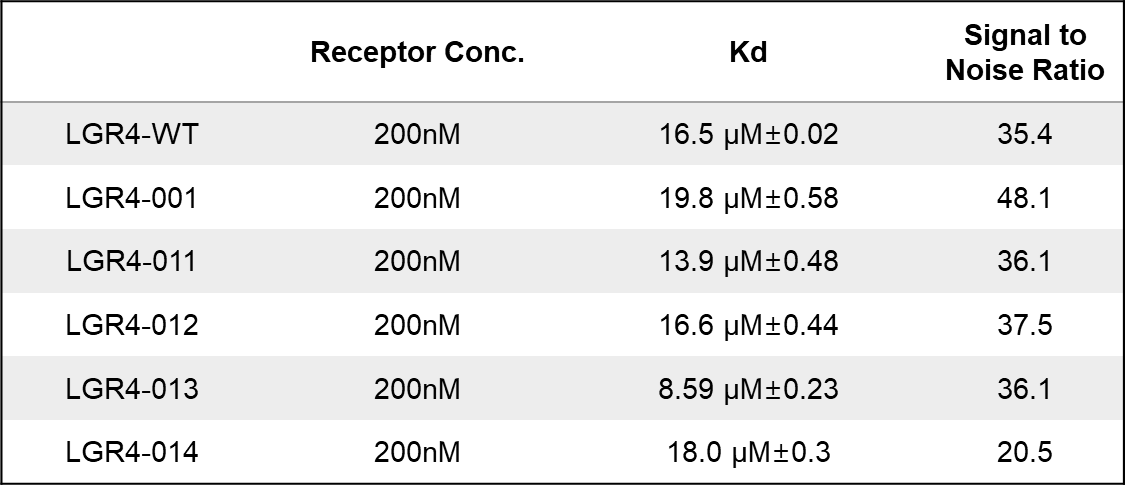


S. 4 Suitability of microscale thermophoresis was presented to determine WT RANKL and MT RANKL binding affinities with LGR4. The data represent the binding affinities (*K*d values) of LGR4 for WT RANKL, 001, 011, 012, 013, and 014. The error bars represent the mean ± S.E. for each data point calculated from three independent thermophoresis measurements. The concentration of WT RANKL, 001, 011, 012, 013, and 014 used in the titration experiments ranged from 11.5 nM to 50 μM, and the concentration of the labeled LGR4 was constant at 200 nM.


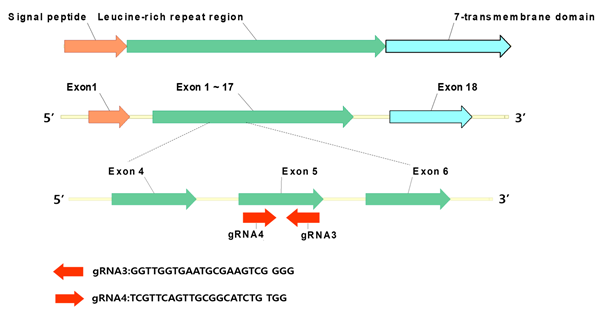
S.5 Schematic diagram of RAW 264.7/LGR4 CKO cell generated using CRISPR-CAS9. The total sequence and matched location of Lgr4 in exons and the location of gRNA in exon 5 using CRISPR/Cas9 methods are presented.


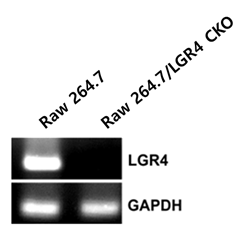


S.6 mRNA expression using RT-PCR in RAW 264.7/LGR4 CKO cells and control RAW 264.7 cells. The deletion of Lgr4 in RAW 264.7/LGR4 CKO cells was detected in exon 5.


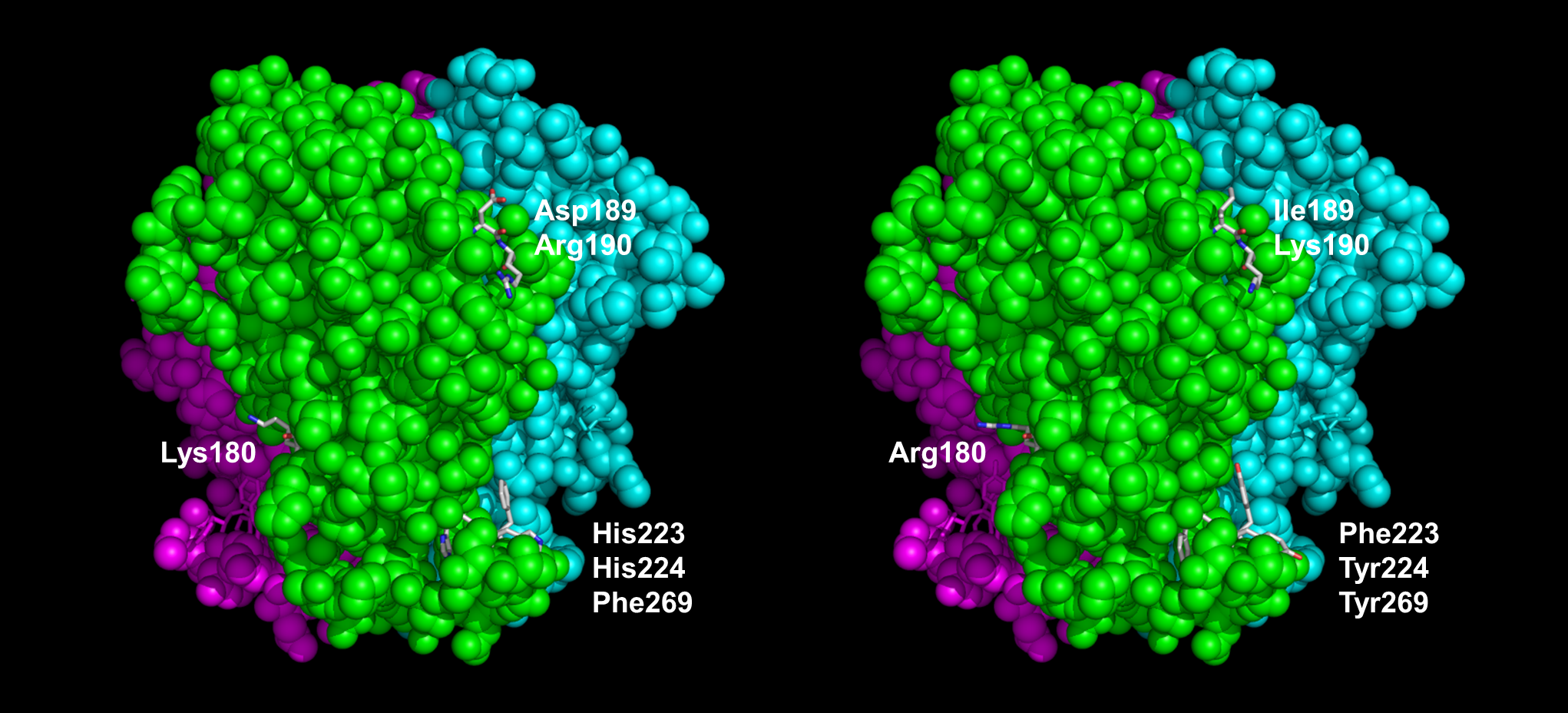


S.7 Models of WT RANKL (Left) and Advanced mutant RANKL (Right) structure based on mouse RANKL structure (PDB ID: 1GIQ). The protein structures between WT RANKL and Advanced mutant RANKL were shown overlapped exactly. Figures were prepared using PyMOL (Ref. De Lano et al, 2002).


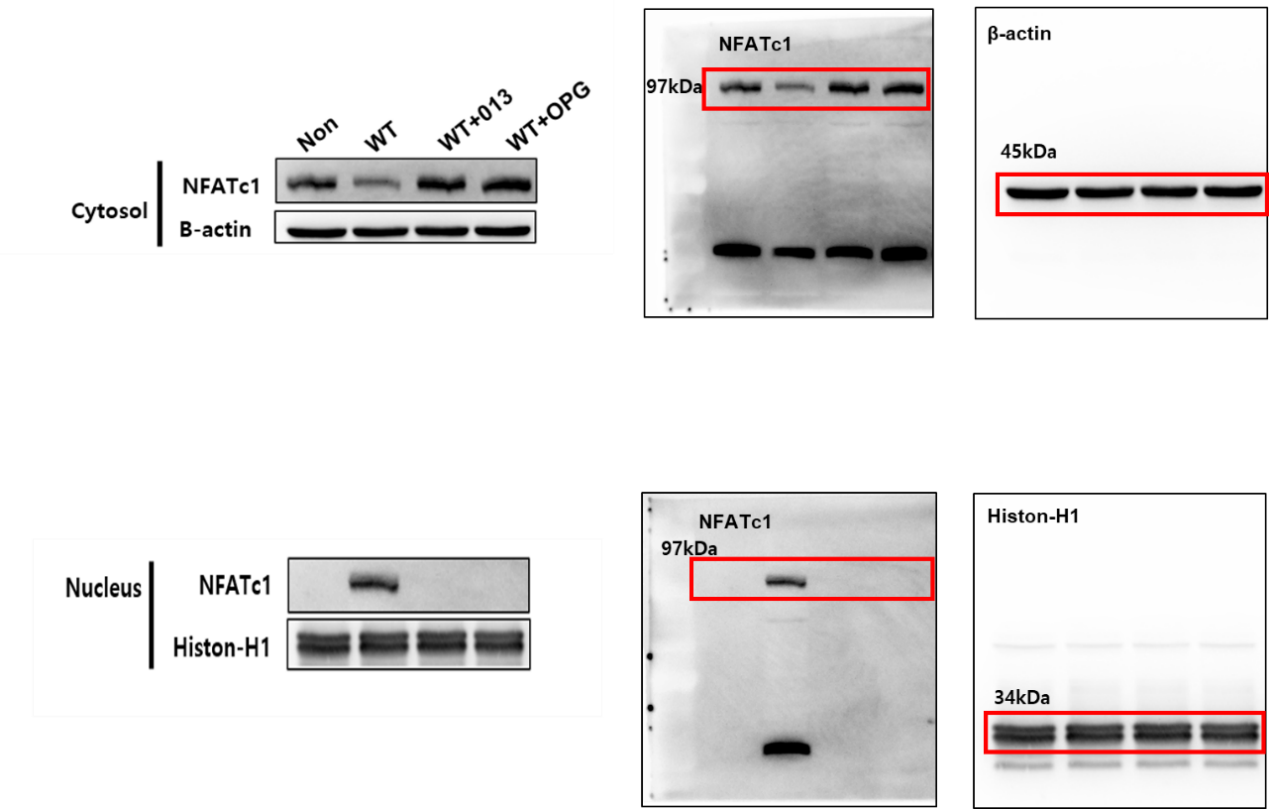


S.8 The original Western blot of Fig. 2D, where the protein molecular weights were labeled.


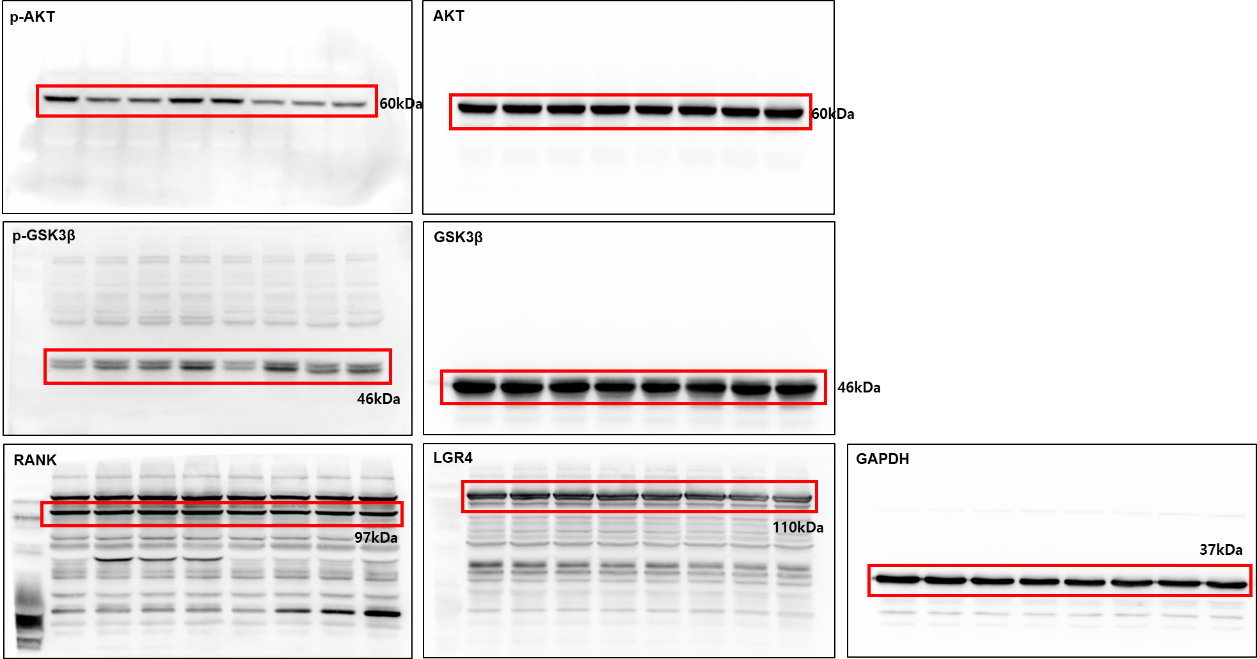


S.9 The original Western blot of Fig. 2F, where the protein molecular weights were labeled.


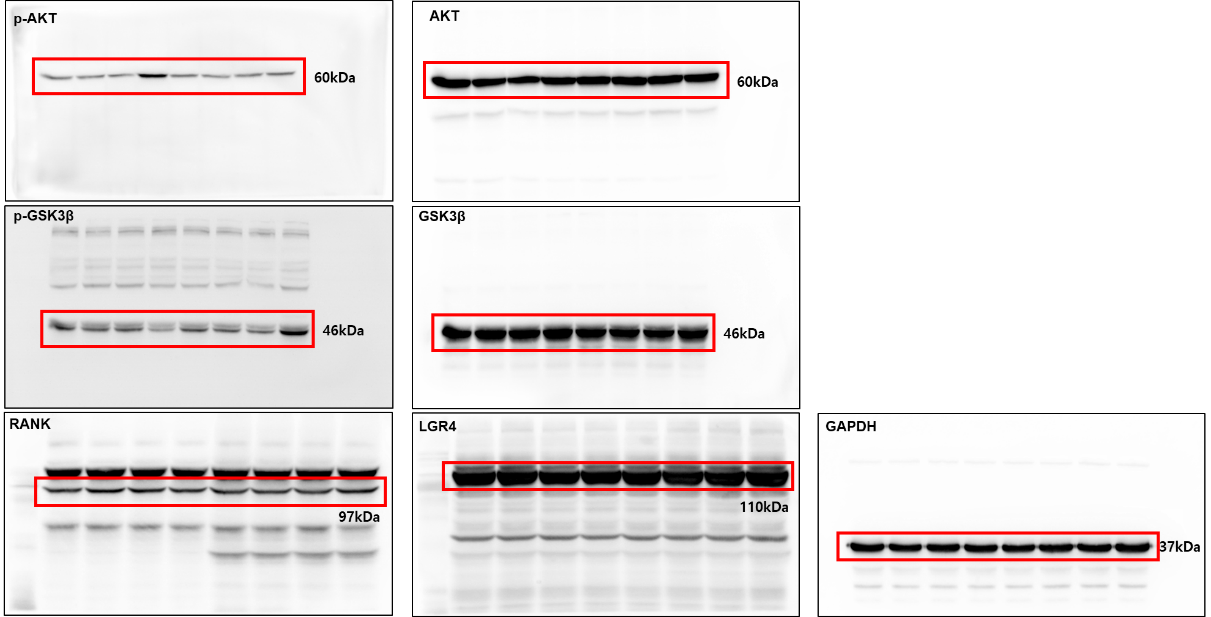


S.10 The original Western blot of Fig. 2J (left, top), where the protein molecular weights were labeled.


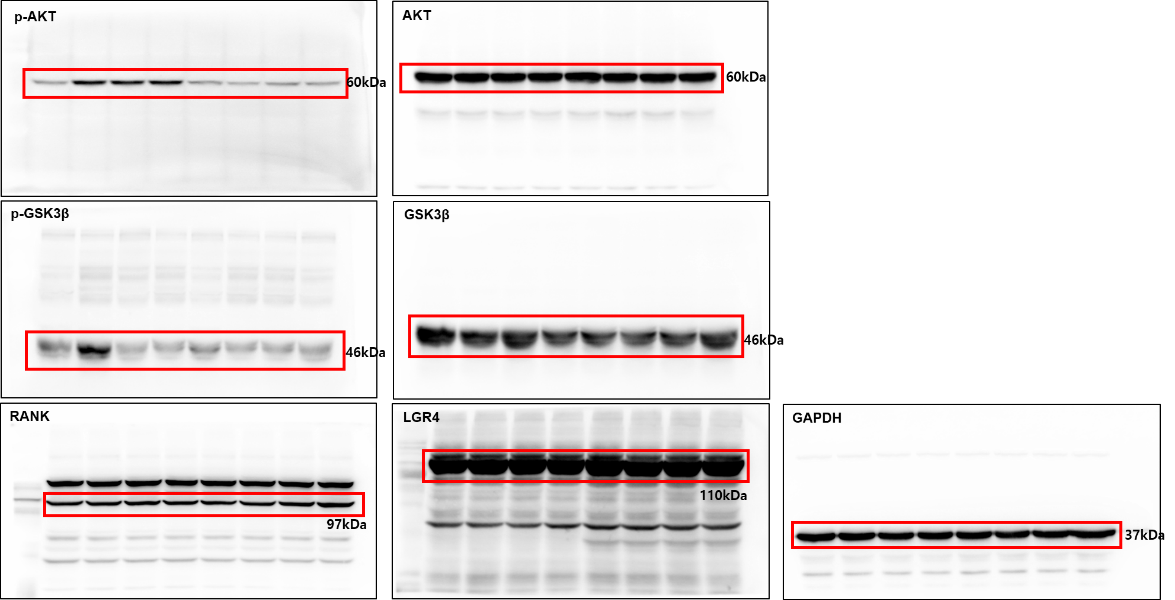


S.11 The original Western blot of Fig. 2J (right, top), where the protein molecular weights were labeled.


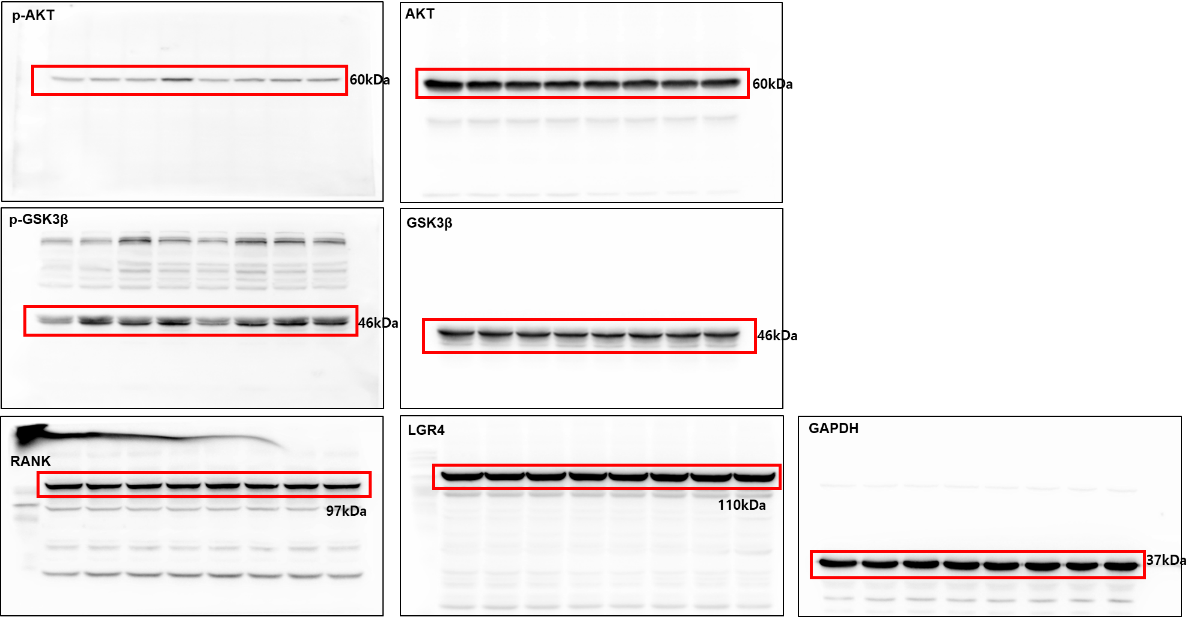


S.11 The original Western blot of Fig. 2J (left, bottom), where the protein molecular weights were labeled.


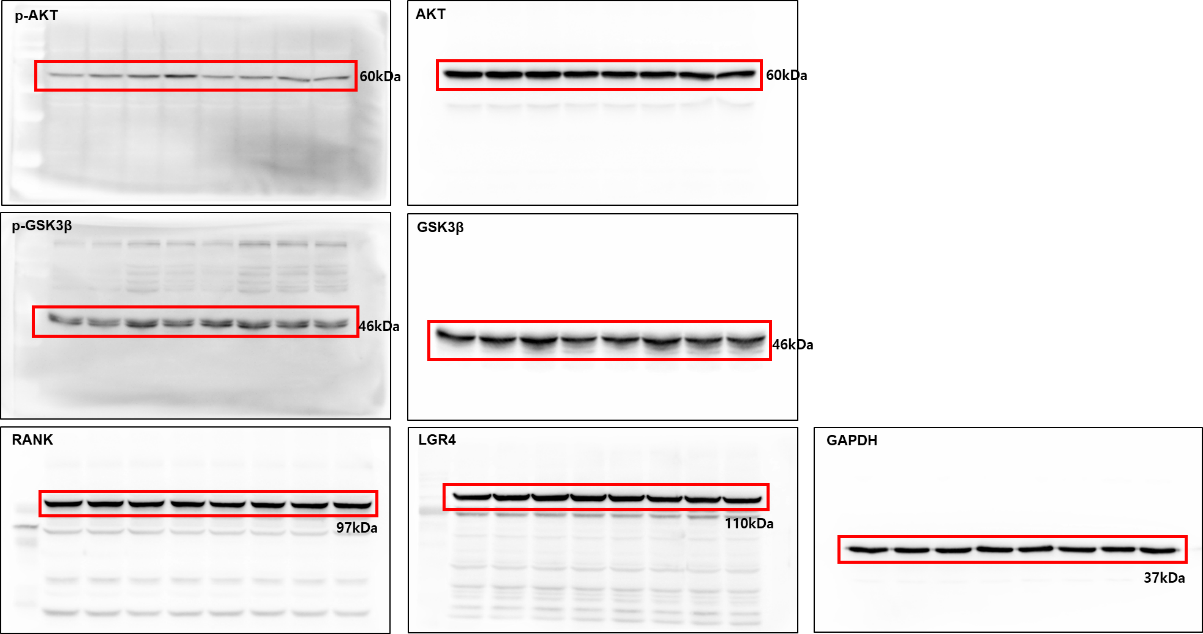


S.12 The original Western blot of Fig. 2J (right, bottom), where the protein molecular weights were labeled.
